# Magnetic properties of FeGa/Kapton for flexible electronics

**DOI:** 10.1038/s41598-022-21589-3

**Published:** 2022-10-19

**Authors:** Gajanan Pradhan, Federica Celegato, Gabriele Barrera, Elena Sonia Olivetti, Marco Coisson, Jan Hajduček, Jon Ander Arregi, Ladislav Čelko, Vojtěch Uhlíř, Paola Rizzi, Paola Tiberto

**Affiliations:** 1 Advanced materials and Life science Divisions, Istituto Nazionale di Ricerca Metrologica (INRIM), 10135 Turin, Italy; 2 Chemistry Department and NIS, University of Turin, 10125 Turin, Italy; 3 Central European Institute of Technology (CEITEC), Brno University of Technology, Brno, 61200 Czech Republic

**Keywords:** Magnetic properties and materials, Surfaces, interfaces and thin films

## Abstract

Flexible materials have brought up a new era of application-based research in stretchable electronics and wearable devices in the last decade. Tuning of magnetic properties by changing the curvature of devices has significant impact in the new generation of sensor-based technologies. In this work, magnetostrictive FeGa thin films have been deposited on a flexible Kapton sheet to exploit the magneto-elastic coupling effect and modify the magnetic properties of the sample. The FeGa alloy has high magnetostriction constant and high tensile strength making its properties susceptible to external stress. Tensile or compressive strain generated by the convex or concave states influence the uniaxial magnetic anisotropy of the system. Low temperature measurements show a hard magnetic behavior and the presence of exchange-bias effect after field cooling to 2 K. The results obtained in this study prove essential for the development of flexible electronics.

## Introduction

Flexible electronics has manifested exploration and development of potential energy efficient technologies like ultrathin sensors, actuators, wearable electronics and new generation straintronic devices in recent years^1–13^. The ability of these electronics to bend, stretch and wrap makes them advantageous over conventional electronics. In this context, interest is growing for engineering of magnetostrictive thin films on flexible substrates for widespread applications where the magnetic properties can be tuned by means of mechanical stress generated due to flexing^14–17^. Among the highly magnetostrictive materials, extensive research has been performed on rare earth-3d transition metal alloys like $\hbox {Tb}_x$ $\hbox {Dy}_{1-x}$ $\hbox {Fe}_2$ (Terfenol-D) having high magnetostriction constant at room temperature ($\sim$ 2000 ppm) and low magneto-crystalline anisotropy. However, the low ductility of these materials have limited its ability to be used in harsh and tensile applications’ environments^18–20^. On the other hand, Iron Gallium (FeGa) has proven to be efficient material for use in actuator and sensor technologies owing to its high tensile strength, high magnetostriction constant (up to $\sim$ 350 ppm at moderate magnetic fields of $\sim$ 10 mT) and soft magnetic behavior^21–23^. The magnetocrystalline anisotropy of FeGa has been reported to be comparatively lower than Terfenol-D which favours change of magnetic properties at low stress fields^24,25^. Fabrication and characterization of magnetostrictive FeGa thin films for industrial applications has gained research interest in the past decade. Epitaxial FeGa thin films with varied composition ($\hbox {Fe}_{1-x}$ $\hbox {Ga}_x$) prepared on ZnSe/GaAs(100) substrates were first studied by Eddrief et al.^26^. The magnetic properties in these thin films were observed by Barturen et al.^27^ revealing the formation of striped magnetic domains. Fin et al. further investigated the in-plane rotation of stripe domains under the application of magnetic field in 65-nm-thick $\hbox {Fe}_{{80}}$ $\hbox {Ga}_{{20}}$ films^28^.

Mechanical stress in a thin magnetostrictive layer induces a change in orientation of its magnetic moments, termed as the Villari effect. The magnetic layer thus, experiences a stress-induced anisotropy and changes in magnetic domain patterns^29,30^. A simple way to submit a magnetostrictive layer to controlled mechanical stress by means of bending or stretching, is to deposit it on a flexible substrate. The effect of stress-induced anisotropy on magnetic stripe domains has been observed in various materials like $\hbox {La}_{0.7}$ $\hbox {Sr}_{0.3}$ $\hbox {MnO}_3$(LSMO)^31^, $\hbox {Fe}_{{81}}$ $\hbox {Ga}_{{19}}$^32^, FeCoSiB^33^, etc. Stress tunability in these materials is relatively low due to rigid substrates. Similar magneto-mechanical coupling effect was also studied on FeTaN^34^, FeZrN^35^, $\hbox {Co}_{{40}}$ $\hbox {Fe}_{{40}}$ $\hbox {B}_{{20}}$^36^, Co^37^ and Co/Pt^38^ thin films deposited on flexible substrates. However, FeGa possesses a much higher magnetostrictive constant as compared to other magnetic materials and hence it is promising for the design of flexible devices and stretchable electronics. Dai et al. has investigated the rotation of stripe domains in $\hbox {Fe}_{{81}}$ $\hbox {Ga}_{{19}}$ films grown on flexible PET (PolyEthylene Terephthalate) substrates by tailoring the stress induced anisotropy with compressive and tensile strains^39,40^. In exchange-biased FeGa/IrMn grown on flexible substrates, the dependence of exchange bias field as a function of strain percentage has been studied^41^. Studies on high frequency magnetic properties in FeGa/PET samples demonstrate an increase in ferromagnetic resonance frequency with strain^42,43^. However, an in-depth characterization describing the correlation between the structural and magnetic properties of FeGa on flexible substrates has not been presented so far.

In order to understand the magnetostrictive behavior, FeGa layers have been deposited on flexible Kapton substrates. The composition of FeGa has been chosen to be 70:30 (Fe:Ga) due to its high magnetostriction constant having mixed ordered and disordered phases^44,45^. Kapton has been considered due to its temperature stability and excellent mechanical properties. A thorough structural characterization of the as-deposited FeGa/Kapton films has been performed to determine the crystal phases of the deposited layers. The magnetic properties of the films have been investigated using magnetic hysteresis and domain characterization. Influence on magnetic properties of FeGa/Kapton has been studied at room temperature by application of stress. Our study reveals that the $\hbox {Fe}_{{70}}$ $\hbox {Ga}_{{30}}$ layer induces huge change in magnetic anisotropy due to magneto-mechanical coupling and thus exhibiting high magnetostriction. Low temperature measurements of magnetic hysteresis have further been performed to understand the properties of different stress-induced states with reduced thermal agitation. This work contributes to the fundamental research about the magnetostrictive FeGa alloys to be used in the field of flexible electronics.

## Results and discussion

### Morphological properties

**Figure 1:**
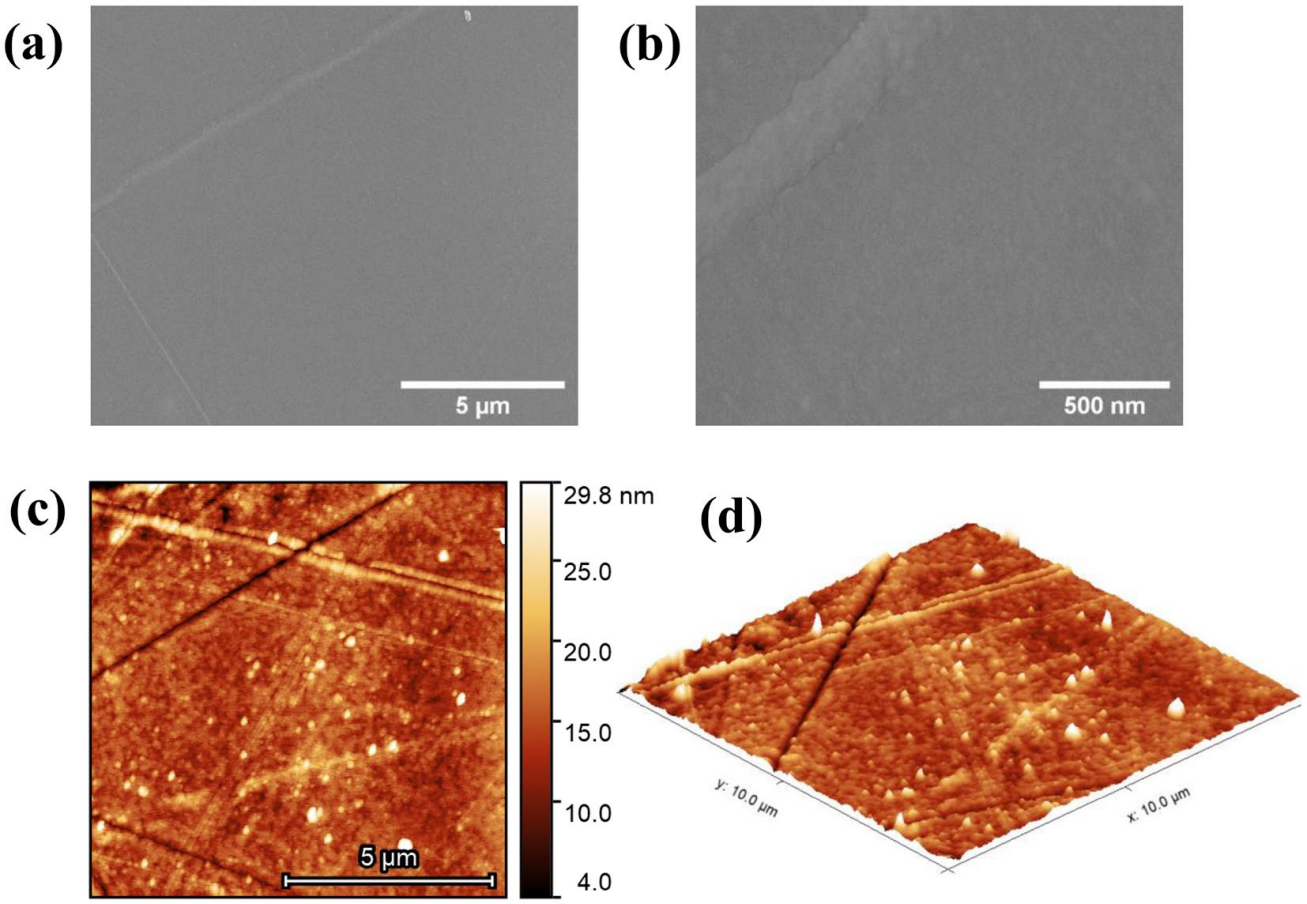
SEM images of the top surface of FeGa (57 nm)/Kapton at scales of 5 $\upmu$m (**a**) and 500 nm (**b**). The topography of the surface measured with AFM is shown in 2D (**c**) and in 3D representation (**d**) for FeGa (28 nm)/Kapton. The RMS surface roughness ($S_q$) is calculated to be 3.03 nm.

Figure 1a shows a wide-field SEM image of the FeGa (57 nm)/Kapton sample. A higher resolution of the sample surface is shown in Fig. 1b which suggests that the film possesses a granular structure. The composition of the FeGa was revealed to be 69.4 %at Fe and 30.6 %at Ga by EDS. This result was in accordance with the concentration determined by ICP-OES i.e. 69.39 %at Fe and 30.61 %at Ga.

Further, surface topography images were recorded using AFM to determine the roughness of the sample surface. Figure 1c,d represent the two-dimensional and three-dimensional surface images obtained with AFM. The images reveal crack lines due to the stress generated at the interface between the magnetic layer and the substrate. The stress can be generated from a bending or flexing of the substrate post sample preparation. The RMS surface roughness ($\hbox {S}_q$) calculated over each point of the scanned area (10 $\upmu$m $\times$ 10 $\upmu$m) is found to be 3.03 nm.

### Structural characterization

**Figure 2:**
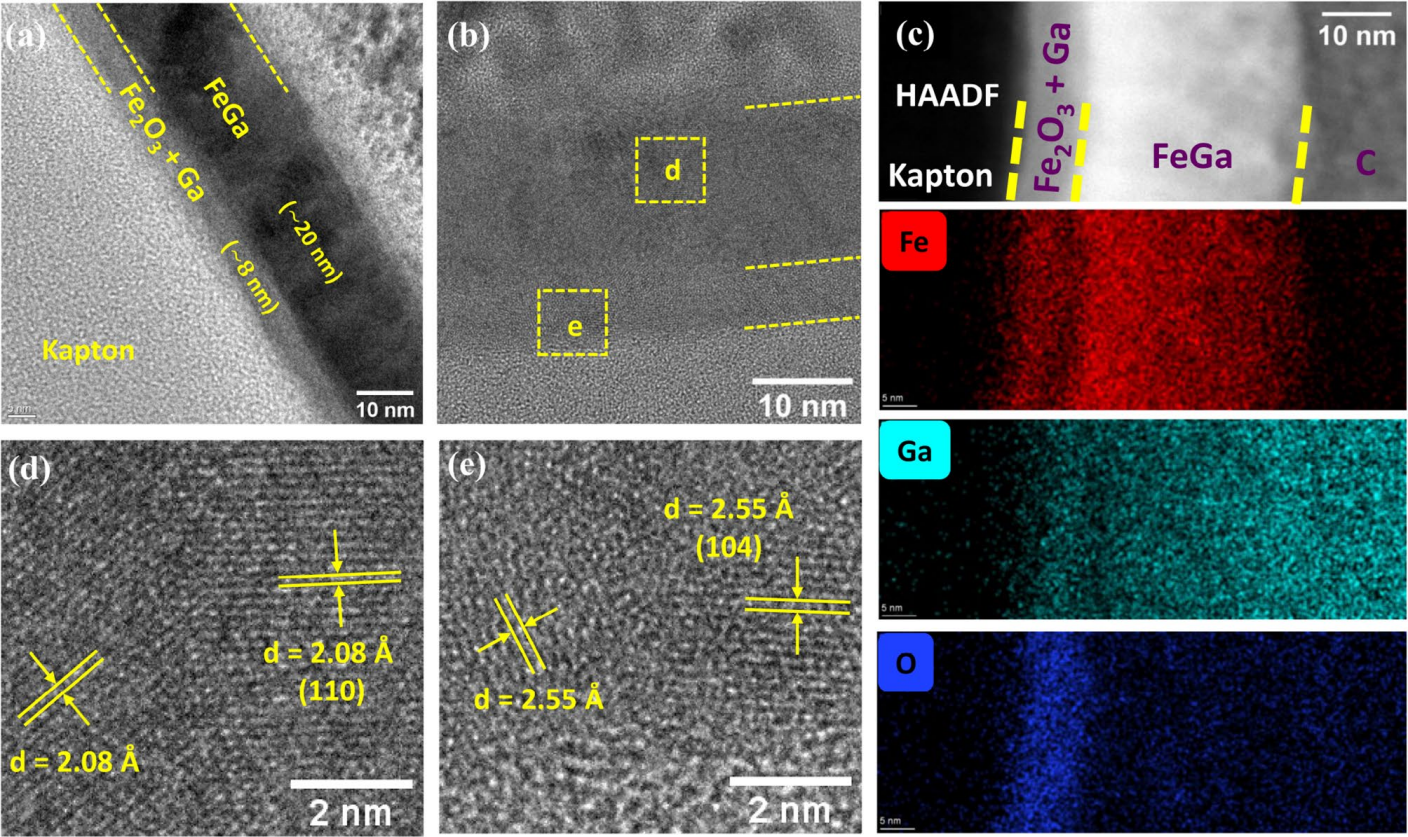
High resolution TEM image of a thin cross-sectional lamella of FeGa (28 nm)/Kapton with low magnification (**a**) and high magnification (**b**). The HAADF and the elemental mapping of the layers are shown in (**c**). (**d**,**e**) Report the measured interplanar distances, which can be indexed as referring to the (110) crystal planes of $\hbox {Fe}_{{70}}$ $\hbox {Ga}_{{30}}$ and (110) crystal planes of $\alpha$-$\hbox {Fe}_2$ $\hbox {O}_3$.

**Figure 3:**
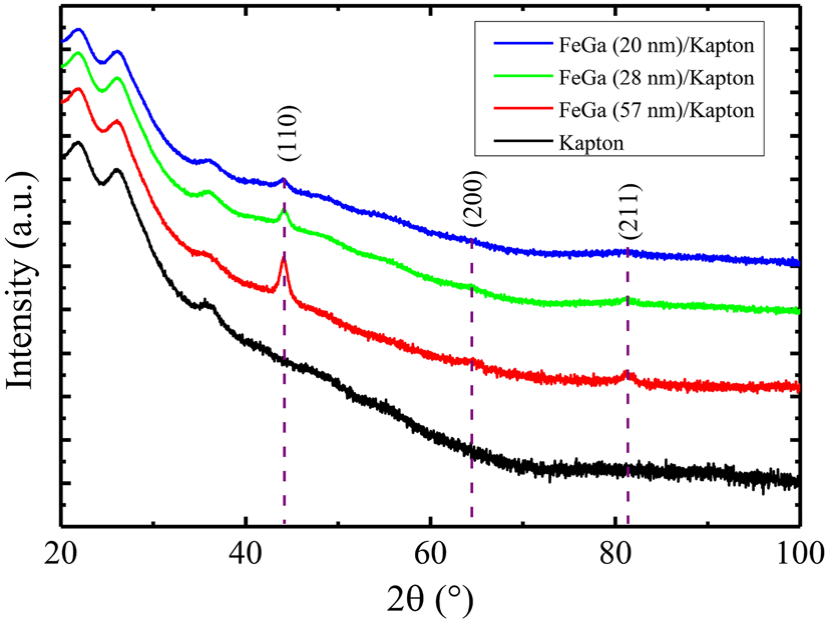
XRD patterns of $\hbox {Fe}_{{70}}$ $\hbox {Ga}_{{30}}$ films of different thickness deposited on Kapton. Red, green and blue graphs corresponds to FeGa thickness of 57 nm, 28 nm, and 20 nm, respectively, and the black graph represents the substrate contribution. The peaks corresponds to the disordered A2 phase of FeGa.

In order to understand the growth structure of FeGa on Kapton, a thin lamella of the FeGa (28 nm)/Kapton sample was analyzed using TEM. The cross-sectional image of the sample is shown in Fig. 2a,b in bright field and high resolution mode, respectively. The amorphous Kapton substrate (light grey colour) is apparent on the left side of Fig. 2a. The FeGa layer is clearly observed with distinguishable interfaces and the thickness is around 20 nm. Interestingly, an additional layer about 8 nm thick, is also visible at the interface between the FeGa layer and the Kapton substrate whose contrast suggests a difference in composition with respect to the FeGa film. Therefore, to understand the chemical composition of present layers, STEM-EDS was performed. The high-angle annular dark field (HAADF) and false colour maps of Fe, Ga and O elemental distribution in the layers is represented by Fig. 2c. The dark region corresponds to the Kapton substrate followed by the magnetic layers and the carbon (C) layer deposited on top during lamella fabrication. There is a considerable presence of Fe (red) and Ga (cyan) atoms in the FeGa layer as compared to other elements. Presence of oxygen atoms (blue) characterizes the interfacial layer between FeGa and Kapton, where the relative amount of Fe and Ga atoms is reduced accordingly. The relative percentage of Fe, Ga and O atoms is shown in Supplementary Fig. S1. The distance between different lattice planes is calculated from the HRTEM images as shown in Fig. 2d,e. Interplanar spacing of 2.08 $\text{\AA}$ which corresponds to the (110) crystal planes of $\hbox {Fe}_{{70}}$ $\hbox {Ga}_{{30}}$ is observed in Fig. 2d. This is also confirmed by the (110) peak in XRD positioned at 2$\theta =$ 44.12$^\circ$^46^. Figure 2e represents the interatomic planes for the layer between the FeGa and Kapton. The measured interplanar spacing in this layer is 2.55 $\text{\AA}$ which corresponds to the presence of (110) planes of the $\alpha$-$\hbox {Fe}_2$ $\hbox {O}_3$ layer in our system^47^. The fast Fourier transform (FFT) patterns corresponding to the different layers has been shown in Supplementary Fig. S2. The diffraction rings corresponds to the 2.08 $\text{\AA}$—$\hbox {Fe}_{{70}}$ $\hbox {Ga}_{{30}}$(110) and 2.55 $\text{\AA}$—$\hbox {Fe}_2$ $\hbox {O}_3$(110) planes. The formation of $\hbox {Fe}_2$ $\hbox {O}_3$ layer is presumably due to the presence of oxygen molecules present on the surface of Kapton before the deposition process. Other hypothesis on the origin of the oxygen have been excluded. In fact, a pre-sputtering of FeGa target was performed before every deposition which rules out the possibility that the oxygen molecules coming from oxidation of the target; the absence of oxygen molecules in the chamber atmosphere during the deposition process was confirmed by using a spectrum analyzer. FeGa was also deposited on $\hbox {SiO}_2$/Si substrates using the same deposition conditions and parameters. It was observed that a similar layer of $\hbox {Fe}_2$ $\hbox {O}_3$ was present between the FeGa layer and the $\hbox {SiO}_2$/Si substrate (Supplementary Fig. S3).

The XRD patterns of the FeGa/Kapton samples are shown in Fig. 3. The red, green and blue graphs represent the data recorded for FeGa thickness of 57 nm, 28 nm and 20 nm, respectively. The pattern of the Kapton substrate (black curve) is also reported, for comparison. The diffraction peaks observed in the FeGa/Kapton samples are positioned at 2$\theta$ values of 44.12$^{\circ }$, 64.13$^{\circ }$ and 81.13$^{\circ }$. In order to uniquely assign these peaks to the D0$_3$ phase, it is necessary to detect the superlattice peak that is expected at low angles, because the other reflections are appearing almost at the same position of the disordered A2 phase. It is also worth noting that thin films do not have high diffraction intensities and, as a consequence, the superlattice peak, if there, can be hidden in the background due to its relative intensity below 10% with respect to the principal reflection. Therefore, from the XRD analysis it is possible to infer that a mixture of A2, B2 and D0$_3$ phases is present in the $\hbox {Fe}_{{70}}$ $\hbox {Ga}_{{30}}$ sample, according to what is reported in literature for Fe-29.9 at.%Ga by Xing et al., where by high energy XRD a mixture of A2, B2 and D0$_3$ phases were observed^45,48^. The (110) reflections of FeGa are the most prominent for all samples and their intensities increases with thickness. The XRD peak corresponding to the $\hbox {Fe}_2$ $\hbox {O}_3$ planes could be present at 35.16$^\circ$ which overlaps with the peak arising from the Kapton substrate. It is not prominently visible due to the low scattering factor of the oxide phase and its lower quantity with respect to the FeGa alloy^47^. The peaks observed below 30$^{\circ }$ in all the samples are characteristic of the Kapton substrate since they appear also in the black curve.

### Magnetic characterization

**Figure 4:**
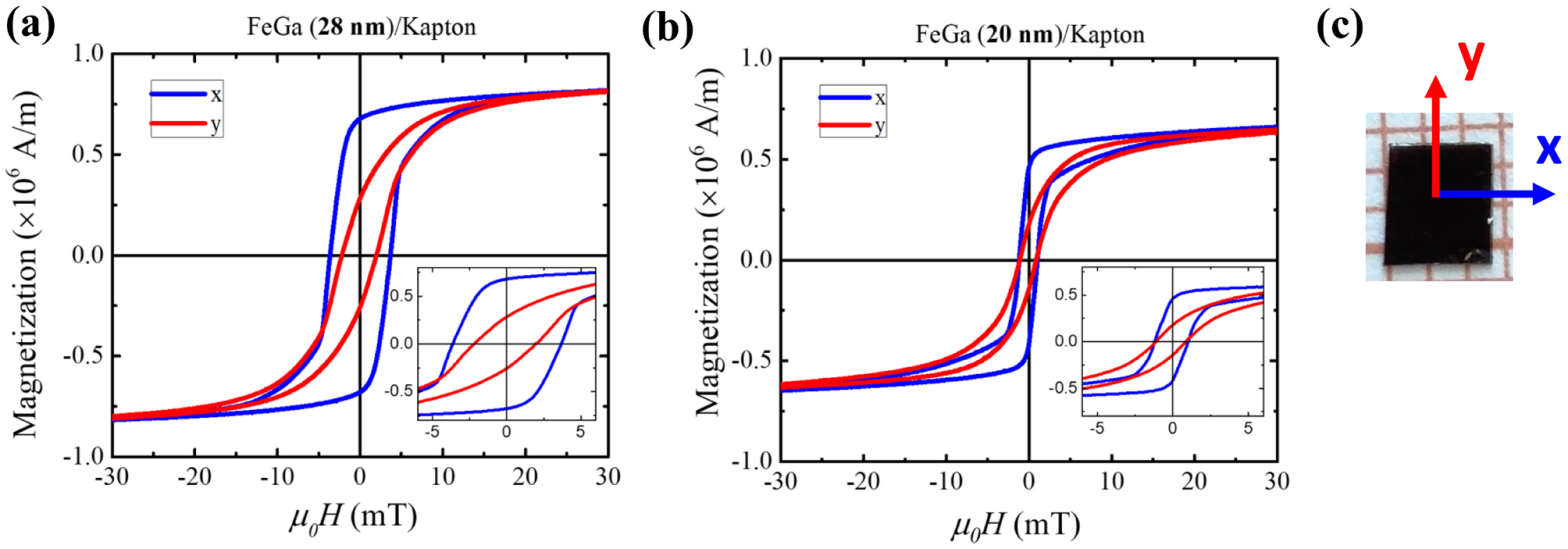
Magnetic hysteresis curves of FeGa/Kapton samples measured with AGFM for different thickness of 28 nm and 20 nm as represented by (**a**) and (**b**), respectively. The blue and red graphs corresponds to the loops measured along x and y (in-plane directions) indicated in (**c**).

Magnetic hysteresis loops ($M-H$ curve) were measured for the FeGa/Kapton samples with application of in-plane magnetic field using AGFM. The samples were saturated at a field of 1 T prior to each measurement. The magnetic hysteresis curves for FeGa thicknesses of 28 nm and 20 nm are shown in Fig. 4a,b, respectively. In-plane hysteresis loop was measured along two perpendicular directions (labeled x and y, see Fig. 4c) to determine the saturation magnetization and to evaluate the magnetic anisotropy of the samples. In practice, the two resulting hysteresis curves were acquired by rotating the sample by 90$^{\circ }$ so as to have x or y direction parallel to the direction of applied field. Figure 4c shows the schematic of the field directions applied to the samples. The hysteresis curves in blue and red colour were measured with field along the x and y directions, respectively. These directions were marked according to a set reference direction during deposition. The hysteresis loops of FeGa (28 nm)/Kapton and FeGa (20 nm)/Kapton shown in Fig. 4a,b, respectively, exhibit a different magnetization behavior along the two perpendicular directions. The x direction loop is more squared as compared to the y direction loop, having higher remanent magnetization ($M_r$) and coercive field ($\mu _0H_c$). The value of $M_r$ and $\mu _0H_c$ along the x and y directions are given in Table 1.

**Table 1:** Remanent magnetization ($M_r$) and coercive field ($\mu _0$ $H_c$) values along the x and y direction of the sample.

	FeGa (28 nm)/Kapton	FeGa (20 m)/Kapton
$M_r$ (x)	0.68 MA/m	0.46 MA/m
$M_r$ (y)	0.28 MA/m	0.18 MA/m
$\mu _0 H_c$ (x)	3.6 mT	1.1 mT
$\mu _0 H_c$ (y)	2 mT	1 mT

The low coercive field values attributes to typical soft magnetic material. The hysteresis loop along the x direction has higher remanence as compared to the loop along the y direction. The magnetic domains in these films tend to stay in-plane due to the shape anisotropy. Therefore, the preferential easy axis (EA) direction is the in-plane x direction and the hard axis (HA) is the in-plane y direction. Untangling the effect of film thickness on magnetic properties is not trivial as the shape of the hysteresis loops depends on various factors such as thickness, grain size, pinning sites, stress induced during deposition, etc. and also by the presence of $\hbox {Fe}_2$ $\hbox {O}_3$ interfacial layer in our case. Hence, the increase in $\mu _0$ $H_c$ and $M_r$ as recorded in Table 1 cannot be uniquely attributed only to variation of a single parameter of FeGa. However, a softening of the magnetic properties with an increase in the thickness of the FeGa film can be deduced from the evaluation of the field ($\hbox {H}_k$) that is the applied field at which the two branches of the hysteresis loop close. In fact, $\hbox {H}_k$ decreases with increasing thickness from 45 to 40 mT for 20 nm and 28 nm thickness respectively. Similar thicknesses of FeGa were also deposited on thermally oxidized Si substrates with an oxide layer of 500 nm. This was performed to understand the magnetic properties of the FeGa thin films due to the alloy itself and not due to the stress effects. The in-plane hysteresis loops of the samples along x and y directions are shown in the Supplementary Fig. S4. It is observed that the FeGa/$\hbox {SiO}_2$/Si samples have lower in-plane anisotropy as compared to the FeGa/Kapton samples. This slight anisotropy can result from the slant deposition of sputtered FeGa atoms on the flat and amorphous $\hbox {SiO}_2$/Si substrate. The same in-plane anisotropic behavior of the FeGa thin film should be observed on the flat and amorphous Kapton substrate. However, a high in-plane anisotropy in the FeGa/Kapton samples is observed, see Fig. 4. The residual stress on the magnetic FeGa layer is due to unavoidable local bending of the Kapton sheet during sample preparation.

**Figure 5:**
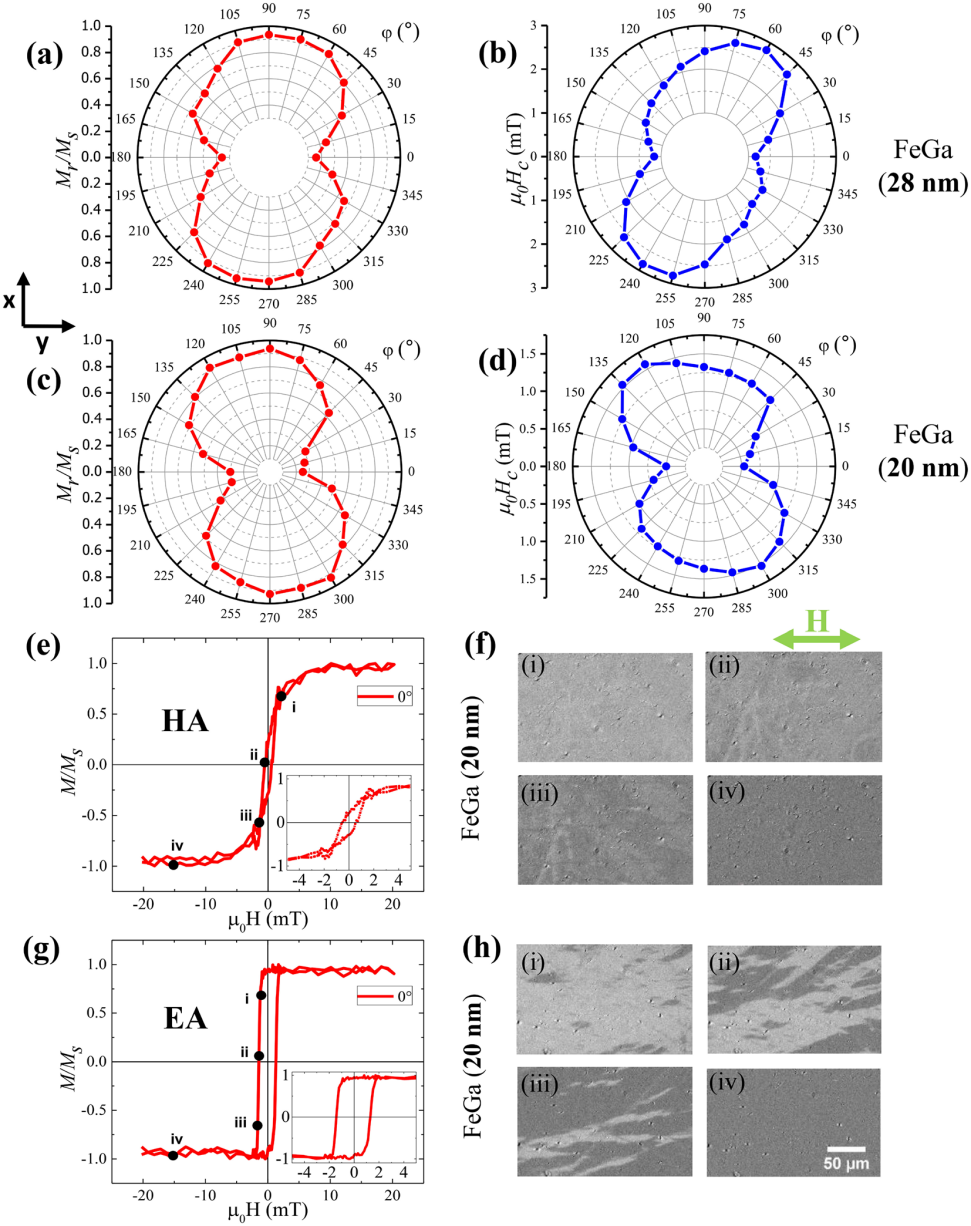
Polar plots of $M_r/M_s$ and $H_c$ as a function of the direction of applied magnetic field, are shown in (**a**) and (**b**) for FeGa (28 nm)/Kapton and in (**c**) and (**d**) for FeGa (20 nm)/Kapton. (**e**) and (**g**) represent the normalized hysteresis curves for the HA and EA, respectively, for the FeGa (20 nm)/Kapton. MOKE images of magnetic domains corresponding to HA and EA are shown in (**f**) and (**g**), respectively, with (i)–(iv) representing the points marked in the hysteresis loops.

Magnetic hysteresis curves were further recorded with MOKE by applying an in-plane magnetic field. The loops were recorded at an interval of 15$^\circ$ rotations of the FeGa/Kapton samples with respect to magnetic field to determine the type of magnetic anisotropy present in the system. These loops specifically correspond to the region of illumination of the polarized light in the Kerr microscope and therefore represent the intensity shift with a change in the magnetic field amplitude. Upon normalization, $M/M_s$ vs $\mu _0H$ is obtained for each of the loops. The $\mu _0H_c$ and $M_r/M_s$ are calculated for all the measured directions. Figure 5a,b and 5c,d represents the 360$^\circ$ polar plot of $M_r/M_s$ and $\mu _0H_c$ for FeGa (28 nm)/Kapton and FeGa (20 nm)/Kapton, respectively. It is observed that both samples exhibit uniaxial anisotropic behaviour. The value of $M_r/M_s$ in Fig. 5a is 0.359 at 0$^\circ$ (y direction) and 0.935 at 90$^\circ$ (x direction) which corresponds to the highest and lowest value. In Fig. 5c, the minimum and maximum values are 0.250 and 0.937 at 0$^\circ$ and 90$^\circ$, respectively. The $M/M_s$ vs $\mu _0H$ curve of the 20 nm film along HA (0$^\circ$) and EA (270$^\circ$) are shown in Fig. 5e,g, respectively. The data represent the typical to the ‘S’-shaped and squared-shaped loops for HA and EA in uniaxial anisotropic systems. The magnetic domain images measured with the Kerr microscope are displayed in Fig. 5f,h corresponding to the HA and EA loops, respectively. The snapshots (i)–(iv) in Fig. 5f are images recorded at 2.3 mT, – 0.5 mT, – 1.3 mT and – 15.2 mT, respectively, as marked in the hysteresis loop. The magnetization reversal is happening via coherent rotation, since the intensity of the image is decreasing and is the darkest at the negative saturation point, Fig. 5f(iv). The magnetic domain images recorded for the EA loop are shown in snapshots (i)–(iv), Fig. 5h which represents the images recorded at − 1.2 mT (near nucleation), − 1.4 mT (near coercivity), − 1.6 mT (near saturation) and − 14.2 mT (saturation point), respectively. Magnetization reversal through domain nucleation and domain wall motion is observed in the sample. Similar results have also been observed in the FeGa (28 nm)/Kapton sample as represented by Supplementary Fig. S5.

### Stress-induced effect

**Figure 6:**
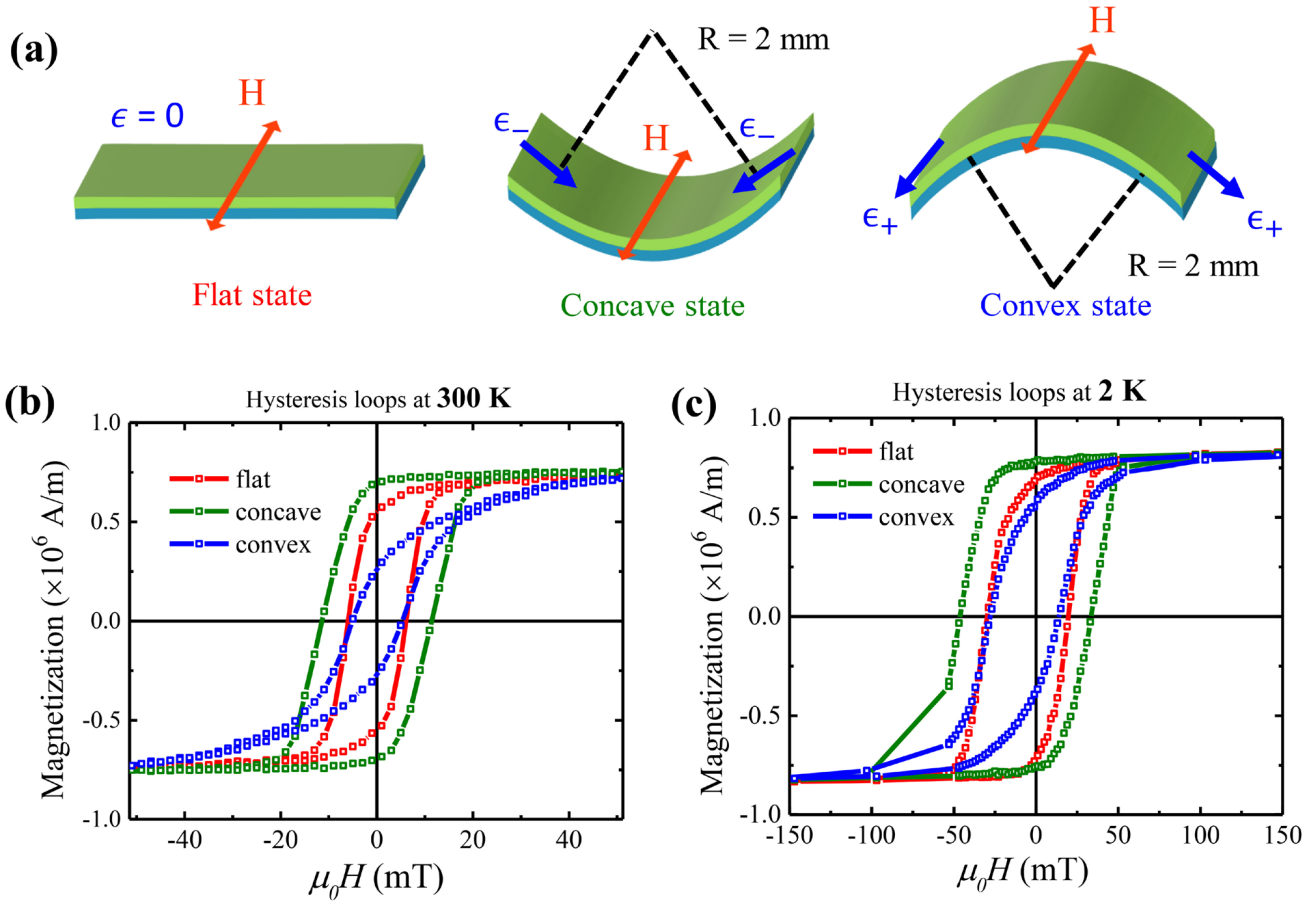
(**a**) Schematic of the flat (no stress), concave (compressive stress) and convex states (tensile stress) obtained by bending of samples; (**b**) and (**c**) magnetic hysteresis loops of FeGa (28 nm)/Kapton sample for flat, concave and convex states, at room temperature (300 K) and at low temperature (2 K).

In order to understand the effect of external stress on the FeGa layers, different molds were prepared for application of both tensile and compressive stress. The schematic of the deformation in samples is shown in Fig. 6a. The flat state, the concave state and the convex state correspond to no stress, compressive stress and tensile stress, respectively. The strain induced on the FeGa film can be determined using the formula^36^:

1 $$\begin{aligned} \varepsilon _-= & {} -t/(2R-t) \end{aligned}$$

2 $$\begin{aligned} \varepsilon _+= & {} t/(2R+t) \end{aligned}$$

where ‘$\varepsilon _-$’ and ‘$\varepsilon _+$’ represent the compressive and tensile strain, respectively, ‘*t*’ is the total thickness of the deposited film and the substrate and ‘R’ is the radius of curvature of bending, which was kept at 2 mm during the measurement. The calculated strain for the convex state is + 2.44% and for the concave state is − 2.56%. At 2 K, thermal strains are also generated in the FeGa layer. The thermal coefficient of $\hbox {Fe}_{79.5}$ $\hbox {Ga}_{20.5}$ as reported previously is nearly 12–14 ppm/$^\circ$C which will generate a thermal contraction of − 0.35% at 2 K^49^. Also, due to the mismatch in thermal coefficients of FeGa and Kapton, stress will be generated at the interface. The strain amounting to this stress in the FeGa layer can be calculated as $\varepsilon _{FeGa}=\Delta T\cdot \Delta \alpha =+0.23\%$. The resulting strain generation in FeGa layer at 2 K are + 2.32% in convex state and − 2.68% in concave state. Similar percentages of strain has been reported in flexible magnetic films like FeGa/IrMn and CoFeB/Kapton films^41,50^. The field is applied in the plane of the sample along the EA and perpendicular to the strain axis to ensure that the field lies in the plane for each point on the sample. Figure 6b shows the in-plane hysteresis loops measured with SQUID-VSM at room temperature (300 K) for flat, concave and convex configurations, which are represented by the red, green and blue curves, respectively. The values of $M_r$ and $\mu _0 H_c$ in the concave, flat and convex states are shown in Table 2. It is observed that the value of remanent magnetization $M_r$ increases with decreasing $\varepsilon$ (concave state) due to compressive strain and decreases with increasing $\varepsilon$, due to tensile strain (convex state). The hysteresis curve in the convex state (blue graph) is similar to the HA hysteresis curve of the FeGa layer shown in Fig. 4a. The value of $M_r$ of both curves is nearly 0.27 $\times$ 10$^{6}$ A/m. The $\mu _0 H_c$ increases for the negative strain applied and decreases for the positive strain. Dependence of coercive field as a function of strain percentage has been reported by Swartzendruber et al. for low carbon steels which shows linear dependence of $\mu _0 H_c$ with the squareroot of strain percentage^51^. The application of stress changes the orientation of magnetic moments along the direction of elongation of sample. This rotates the magnetic EA along the direction perpendicular to applied field in the convex state. Hence, the hysteresis loop observed is similar to HA loop of flat state. In the concave state, the compressive stress forces the spins to align more along the direction of EA, thereby reinforcing the original EA direction. The magnetization vector follows the elongation of the crystal lattice. Similar changes of magnetic anisotropy due to external stress have also been reported in CoFeB by Tang et al.^36^. Rotatable magnetic anisotropy behaviour has also been studied by Coisson et al.^52^. The magnetic anisotropy field of the magnetostrictive FeGa layer gets drastically affected by the impact of stress generated evenly through the overall thickness of the FeGa layer due to bending which is termed as the magneto-mechanical coupling effect. Such effects are not expected from the interfacial layer containing $\alpha$-$\hbox {Fe}_2$ $\hbox {O}_3$ phases due to very low mangetostriction constant^53^.

**Table 2:** Remanent magnetization ($M_r$) and coercive field ($\mu _0$ $H_c$) values for the concave, flat and convex states of FeGa (28 nm)/Kapton sample at 300 K and 2 K.

	Temperature	Concave	Flat	Convex
$M_r$	300 K	0.7 MA/m	0.55 MA/m	0.27 MA/m
$\mu _0 H_c$	300 K	11.4 mT	6 mT	5 mT
$M_r$	2 K	0.78 MA/m	0.68 MA/m	0.55 MA/m
$\mu _0 H_c$	2 K	40.9 mT	24.5 mT	20.5 mT

Low-temperature measurements of hysteresis curves were performed down to T $\sim$ 2 K in order to understand the magnetic behaviour of the samples in conditions of reduced thermal fluctuations. Figure 6c shows the magnetic hysteresis curves of FeGa (28 nm)/Kapton sample measured at 2 K. The sample configurations during measurements performed at 300 K and 2 K remain exactly the same. We observe that the value of $\mu _0H_c$ and $M_r$ increases for each of the strained states as reported in Table 2. Exponential dependence of $\mu _0H_c$ on temperature is shown in Supplementary Fig. S6a. The dependence of saturation magnetization ($M_s$) as a function of temperature is shown in Supplemntary Fig. S6b. Similar results have also been observed in the FeGa (20 nm)/Kapton sample as represented by Supplementary Fig. S7. The increase of $\mu _0H_c$ and $M_r$ with the lowering of temperature depicts that the system requires more energy for the reversal of magnetic moments across the energy barrier. This energy difference is compensated by the Zeeman energy which is associated with the application of higher magnetic field. The hard magnetic behavior of magnetic bulk materials and thin films at low temperatures well reported^54–56^.

**Figure 7:**
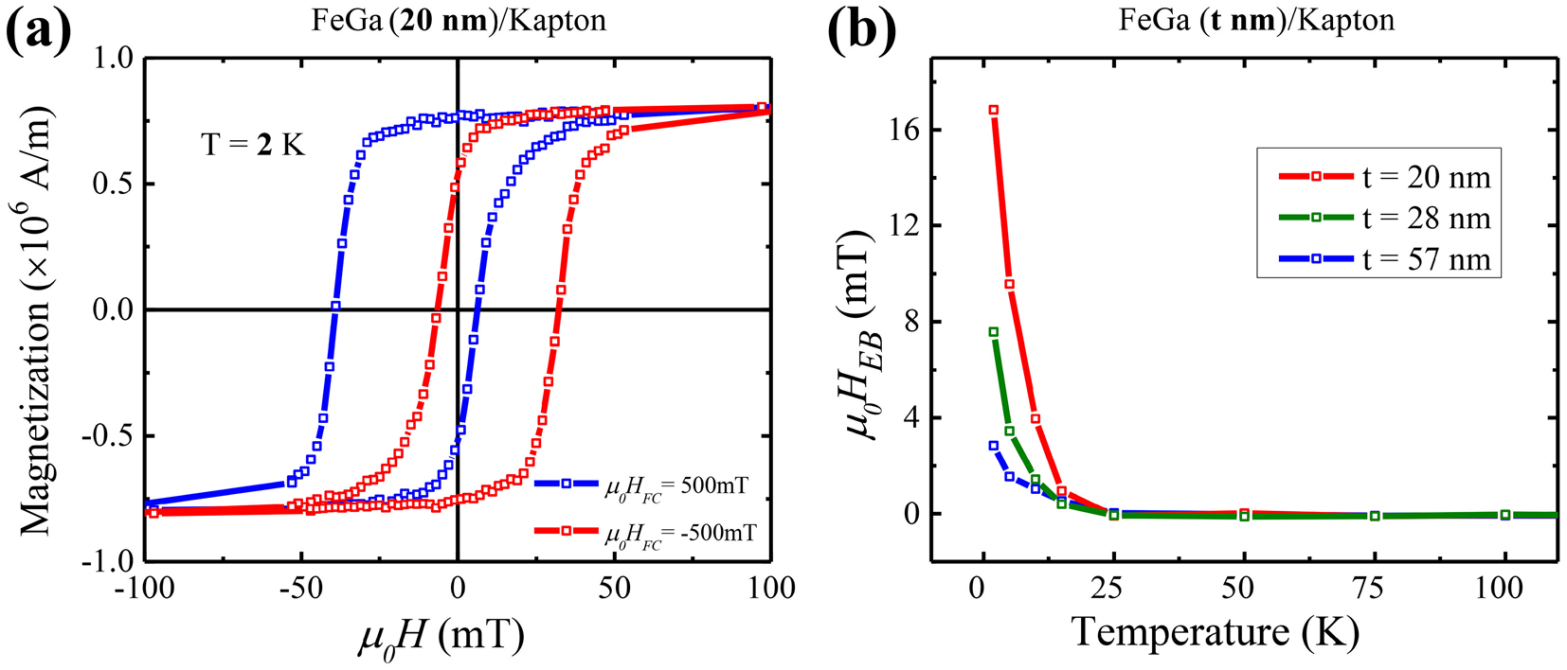
Magnetic hysteresis curves recorded at 2 K after field cooling for FeGa (20 nm)/Kapton is shown in (**a**). The blue and red curves represent the curves for cooling fields of + 500 mT and − 500 mT, respectively. Exchange bias field ($\mu _0 H_{EB}$) as a function of temperature is shown in (**b**) for FeGa thickness of 20, 28 and 57 nm.

### Exchange-bias effect

The magnetic hysteresis curves at 2 K were measured with SQUID after magnetic field cooling at ±500 mT. Figure 7a represents the hysteresis curves after the field-cooling procedure at $\mu _0 H_{FC}$ = 500 mT (blue graph) and at $\mu _0 H_{FC}$ = − 500 mT (red graph). It is clearly observed that there is a symmetrical shift of the hysteresis loop in opposite directions. This indicates the presence of exchange bias in the FeGa/Kapton samples with different thickness. The exchange bias field ($\mu _0 H_{EB}$) is calculated as a function of temperature for different thicknesses of FeGa as shown in Fig. 7b. The $H_{EB}$ is calculated for each hysteresis curve by

3 $$\begin{aligned} H_{EB}= \frac{H_a+H_b}{2} \end{aligned}$$

where $H_a$ and $H_b$ represent the coercive field during the increasing and decreasing field branches of the loop. The final value of $\mu _0 H_{EB}$ is the average of the $\mu _0 H_{EB}$ calculated for ±500 mT. At 2 K, the highest $\mu _0 H_{EB}$ (16.8 mT) is recorded for the lowest thickness (20 nm) and the lowest value (2.9 mT) is found for the FeGa thickness of 57 nm. We attribute the origin of the exchange bias effect in our FeGa/Kapton samples to the formation of an antiferromagnetic $\alpha$-$\hbox {Fe}_2$ $\hbox {O}_3$ interstitial layer during the early stages of thin-film depositions^57,58^. The thickness of this layer is approximately 8 nm as observed in the TEM image in Fig. 2. With higher thickness of the FeGa layer, the size of the magnetic hysteresis loop shift is mitigated due to the interfacial nature of the exchange bias phenomenon, as shown in Fig. 7b^59^.

## Conclusion

The effect of stress brings up a significant change in the magnetic properties of magnetostrictive FeGa thin films due to mechanical magneto-elastic coupling. The preparation of FeGa thin films leads to a mixed ordered and disordered phase for the 70:30 (Fe:Ga) composition. The polycrystalline nature of metallic FeGa on the flexible substrate and the surface topography with minimal roughness makes our samples suitable to investigate their magnetic and magneto-elastic properties. Uniaxial anisotropy is observed in the samples with magnetization reversal occurring through domain wall motion for the easy axis directions. The magnetic anisotropy of FeGa/Kapton samples is higher as compared to the FeGa/$\hbox {SiO}_2$/Si samples due to the residual stress generation and strain transfer to the FeGa layer from Kapton. The curvature of the FeGa/Kapton samples determining the generation of compressive or tensile strain, greatly modifies the in-plane anisotropy direction of the samples. We also showed that the EA can be turned into the HA of the sample by application of tensile strain while the compressive strain reinforces the EA in the original direction.

The low temperature in-plane hysteresis loops measured at 2 K depict a hard magnetic behaviour of FeGa. The increase in magnetic coercivity of both the flat and stressed states signifies the additional Zeeman energy required to compensate the decrease of thermal energy with respect to room temperature. The field-cooled magnetic hysteresis curves at low temperatures reveal the presence of exchange-bias effect in our system due to the formation of an antiferromagnetic $\hbox {Fe}_2$ $\hbox {O}_3$ layer between the FeGa and Kapton. This effect is more pronounced for lower FeGa thickness. We plan to investigate $\hbox {Fe}_{{81}}$ $\hbox {Ga}_{{19}}$ films in similar conditions since this composition also has high magnetostriction constant with a disordered phase and may display even stronger effects, suitable for exploitation in sensors and actuators applications. Our work elucidates the interplay of stress and magnetic anisotropy in flexible FeGa films and demonstrates their tunability for the field of flexible electronics.

## Methods

$\hbox {Fe}_{{70}}$ $\hbox {Ga}_{{30}}$ thin films were deposited on Kapton (polyimide) substrates of 100-$\upmu$m thickness at room temperature by RF sputtering in a high vacuum chamber. The source target used here has the same composition. The base pressure of the chamber was 10$^{-7}$ mbar and the deposition pressure was kept at 1.$2 \times 10$ $^{-2}$ mbar. No external magnetic field was applied near the substrate during the deposition. Three different samples were prepared with FeGa thickness of 20 nm, 28 nm and 57 nm. The film thickness was determined by measuring the step height of FeGa using an atomic force microscope (AFM).

Room-temperature magnetic hysteresis curves were measured using alternating gradient field magnetometer (AGFM) by applying magnetic field along the in-plane directions of the sample. For comparison of hysteresis curves at room temperature and low temperatures (down to 2 K), superconducting quantum interference device - vibrating sample magnetometer (SQUID-VSM, MPMS3) by Quantum Design Ltd was used. Field-cooled measurements were performed by applying a constant DC field while decreasing the temperature. In addition, angle-dependent magnetic hysteresis loops were measured using magneto-optical Kerr effect (MOKE) microscopy from Evico magnetics equipped with a rotatable sample stage in longitudinal mode. This setup was also used to observe the in-plane magnetic domains formed during magnetization reversal.

X-ray diffraction (XRD) was used to determine the phase composition of the samples with a PANalytical X’Pert Pro MPD system. To limit the X-ray penetration depth in order to obtain a stronger signal from the film, measurements were performed with a fixed glancing angle of incidence of 1 using Cu $\hbox {K}_\alpha$ radiation in a pseudo-parallel beam configuration. Electron transparent TEM lamellae of the samples were fabricated using focused ion beam/scanning electron microscope (FIB/SEM) by FEI Helios NanoLab 660. The lamellae were imaged using a FEI Titan Themis 60-300 Cubed high resolution (scanning) transmission electron microscope (HR-TEM) at 300 kV equipped with SUPER-X energy dispersive X-ray spectroscopy (EDS) spectrometer by FEI. The surface morphology and elemental composition of deposited FeGa films were investigated with a scanning electron microscope (FEI Inspect F) equipped with an energy dispersive detector (SEM-EDS). Fe and Ga concentrations were also determined by inductively coupled plasma–optical emission spectroscopy (ICP-OES–Optima 7000 DV Perkin Elmer) equipped with a PEEK Mira Mist nebulizer, a cyclonic spray chamber and an Echelle monochromator. The wavelengths were 238.204 and 417.206 nm for Fe and Ga, respectively. The concentration values were averaged on the basis of three instrumental measurements. Acid digestion in a microwave oven (Milestone MLS-1200 MEGA) was chosen as dissolution procedure. Sample aliquots were treated with 5ml of aqua regia in tetrafluoromethoxyl (TFM) bombs. Four heating steps of 5 min each (250, 400, 600, 250W power respectively), followed by a ventilation step of 25 min, were applied. At the end of the full treatment, the samples appeared completely dissolved but not the silica substrate. Finally, the resulting solutions were diluted to 20 ml with high purity water (HPW).

The surface topography of the samples was recorded with AFM using the Bruker Multimode V Nanoscope 8 setup. Here, MESP magnetic tips (resonant frequency of 75 kHz with Co/Cr reflective coating) were used for acquiring the topography and magnetic signals. The root mean square (RMS) surface roughness ($S_q$) of the scanned area was calculated using the Gwyddion software^60^.

## Supplementary Information

Supplementary Information.

## Data Availability

The raw/processed data required to reproduce these findings are available from the corresponding authors on request.
