# Supplementary material for: Magnetic properties of FeGa/Kapton for flexible electronics
Source: Sci Rep. 2022 Oct 19;12:17503. doi: 10.1038/s41598-022-21589-3 (PMC9582224; doi:10.1038/s41598-022-21589-3)
Supplement: Supplementary file 1 — Supplementary Information. [file 41598_2022_21589_MOESM1_ESM.pdf]

Supplementary Information
Magnetic properties of FeGa/Kapton for flexible electronics

Gajanan Pradhan,*,† Federica Celegato,† Gabriele Barrera,† Elena Sonia Olivetti,† Marco Coisson,† Jan Hajduček,¶ Jon Ander Arregi,¶ Ladislav Čelko,¶ Vojtěch Uhlíř,¶ Paola Rizzi,‡ and Paola Tiberto†

†*Advanced materials and Life science Divisions, Istituto Nazionale di Ricerca Metrologica (INRIM), Strada delle Cacce 91, 10135, Torino, Italy*

‡*Chemistry Department and NIS, University of Turin, via Pietro Giuria, 7, 10125, Torino, Italy*

¶*Central European Institute of Technology (CEITEC), Brno University of Technology, Purkyňova 123, Brno 61200, Czech Republic*

E-mail: g.pradhan@inrim.it

Structural information:

The microstructure analysis of the FeGa/Kapton films has been performed using transmission electron microscopy (TEM). The relative fraction of different atoms present in the layers is represented in Fig. S1(b), corresponding to the HAADF image in Fig. S1(a). The red, cyan and blue curves represent the relative percentage of Fe, Ga and O atoms as a function of position along the scanned area of the lamella. A pronounced relative percentage of O atoms in the layer between Kapton and FeGa indicates the presence of an α-Fe_2O_3 layer.

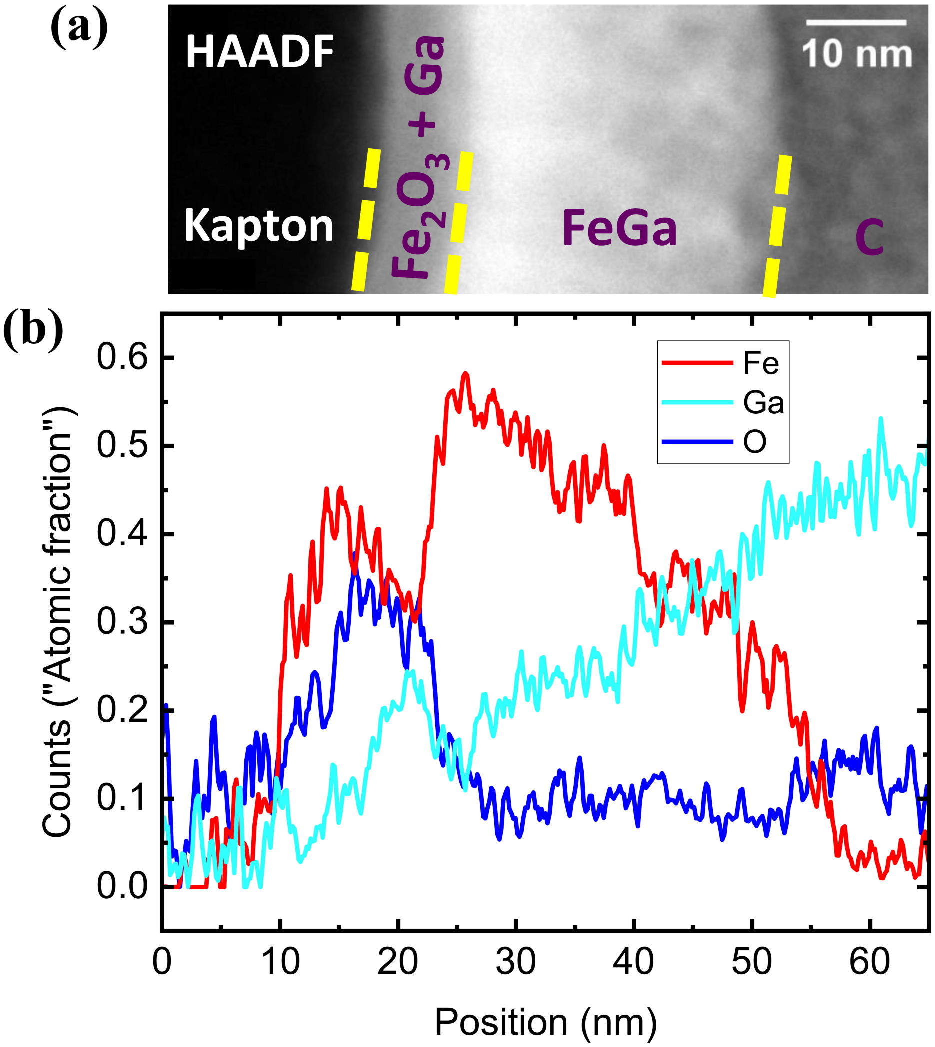

Figure S1: (a) HAADF image of the lamella. (b) Relative atomic percentages of Fe, Ga and O as a function of position in the layers (corresponding to the HAADF image) are represented by red, cyan and blue curves, respectively.

Figure S2(a) represents a cross-sectional image of the FeGa (28 nm)/Kapton sample. The fast Fourier transform (FFT) of different regions (indicated by blue squares) is performed across the layers in order to obtain the corresponding lattice spacing of atoms. The top red square includes the FeGa layer, while the bottom red square includes the Fe_2O_3 layer and a fraction of the Kapton substrate. The FFT of the top and bottom square is shown in Fig. S2(b) and S2(c), respectively. Interplanar distances of 0.208 nm corresponding to (110) - FeGa and 0.255 nm corresponding to (110) - Fe_2O_3 are observed as defined by the red circles.

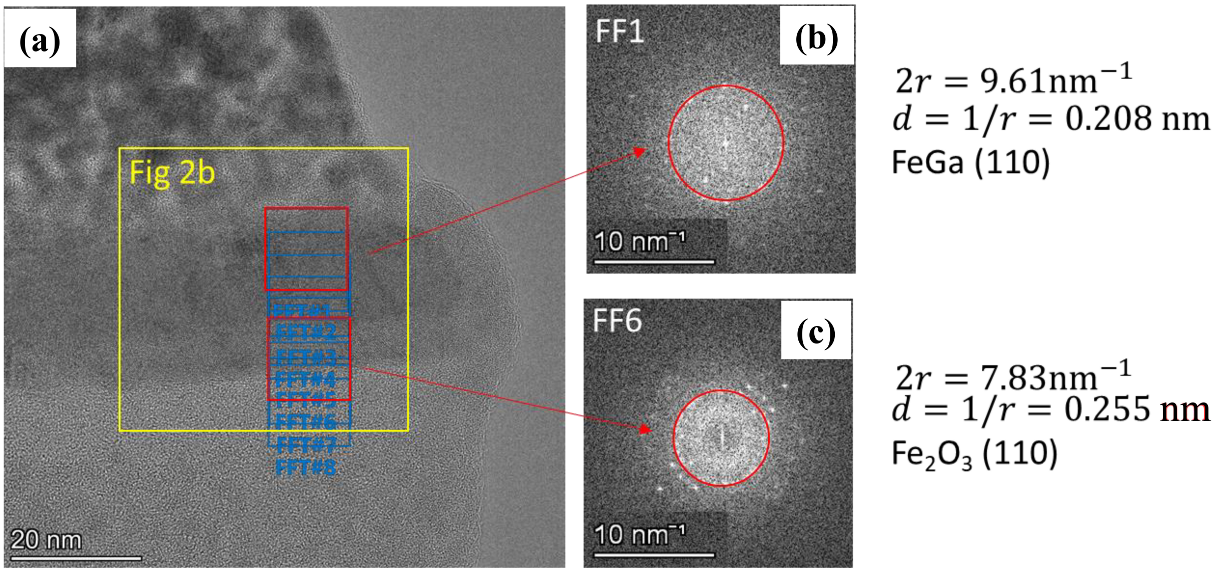

Figure S2: (a) High resolution TEM image of FeGa (28 nm)/Kapton lamella. The FFT images for the FeGa and Fe_2O_3 layers are shown in (b) and (c), respectively. The red circles represent the distance between crystal planes.

A thin lamella of FeGa (28 nm)/SiO_2/Si sample was fabricated and observed in TEM, analogously to the FeGa (28 nm)/Kapton sample. Figures S3(a) and S3(b) represent the images observed in the bright field and high resolution modes, respectively. It has been observed that a similar Fe_2O_3 layer is formed between the SiO_2 and the FeGa layers as in the FeGa/Kapton samples.

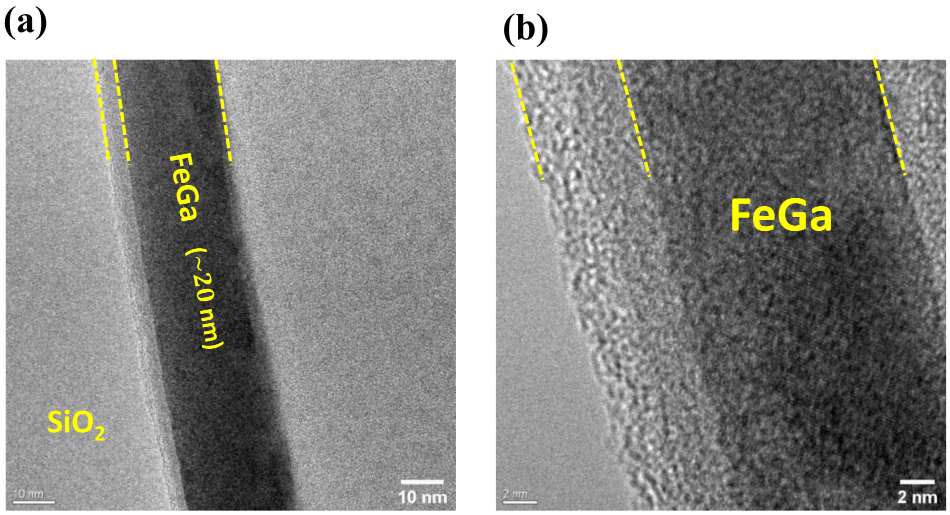

Figure S3: Cross-section TEM images of FeGa (28 nm)/SiO_2/Si lamella in (a) low and (b) high magnification.

Magnetic characterization:

The magnetic hysteresis curves of FeGa/SiO_2/Si samples were measured with an alternating gradient field magnetometer (AGFM). Figures S4(a) and S4(b) represent the hysteresis loops for FeGa thicknesses of 28 nm and 20 nm, respectively. The red and blue curves represent the loops measured along x and y direction (in-plane orthogonal directions) which were marked according to a set reference direction during deposition. For the FeGa (28 nm)/SiO_2/Si, the M_r and $\mu_0 H_c$ are 0.25×10^6 A/m and 0.3 mT along the x direction and 0.16×10^6 A/m and 0.4 mT along the y direction, respectively. For the FeGa (20 nm)/SiO_2/Si, the M_r and $\mu_0 H_c$ are 0.06×10^6 A/m and 0.2 mT along the x direction and 0.21×10^6 A/m and 0.3 mT along the y direction, respectively. The magnetic anisotropy of these samples is low as compared to the FeGa/Kapton samples.

Magnetic hysteresis measurements using magneto-optic Kerr effect (MOKE) were performed for the FeGa (28 nm)/Kapton sample. The normalized hysteresis loops (M/M_s vs $\mu_0 H$ curve) measured along the hard axis (HA) and the easy axis (EA) is shown in Fig. S5(a) and S5(c), respectively. The magnetic domain images for the HA shown in S5(b)(i)-(iv) correspond to the field values of 6.5 mT, -0.2 mT, -1.6 mT and -15 mT, respectively.

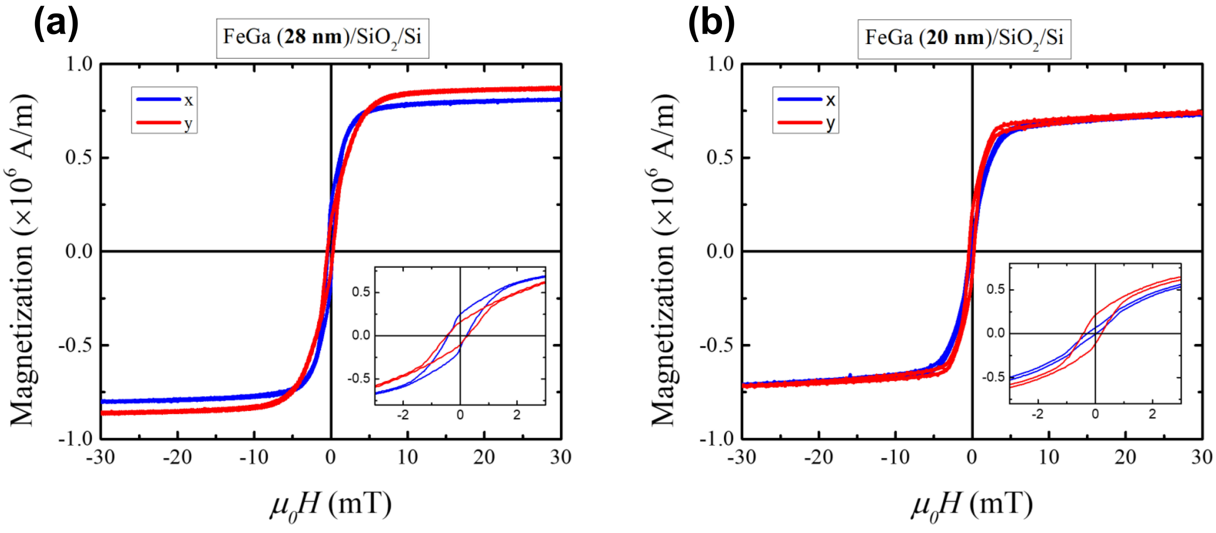

Figure S4: Magnetic hysteresis curves of FeGa/SiO_2/Si samples measured with AGFM for FeGa thicknesses of (a)28 nm and (b)20 nm. The blue and red graphs correspond to the loops measured along x and y directions, referenced in the same way as in FeGa/Kapton samples.

Similarly, S5(d)(i)-(iv) represent the domain images recorded at -2.2 mT, -2.4 mT, -2.6 mT and - 15 mT, respectively. The magnetization reversal occurs via coherent rotation along HA and via domain wall motion in along EA.

Figure S6(a) shows the dependence of coercive field ($\mu_0 H_c$) as a function of temperature. The values of $\mu_0 H_c$ are calculated from hysteresis loops measured with superconducting quantum interference device - vibrating sample magnetometer (SQUID-VSM) at a range of temperature varying from 2 K to 400 K. The $\mu_0 H_c$ is higher at lower temperatures of 2 K - 25 K showing an exponential dependence. The plot of M_s vs temperature is shown in Fig. S6(b). The value of M_s decreases with increase in temperature as the thermal disorder hinders the alignment of moments with the applied magnetic field.

The in-plane magnetic hysteresis measured with SQUID-VSM for flat, concave and convex states for FeGa (20 nm)/Kapton sample are shown in red, green and blue curves respectively at 300 K (Figure S7(a)) and at 2 K (Figure S7(b)). At 300 K, the value of M_r is 0.61 $\times$ 10^6 A/m, 0.23 $\times$ 10^6 A/m and 0.18 $\times$ 10^6 A/m for the concave, flat and convex states, respectively. Similarly, at 2 K, the values are 7.6 $\times$ 10^5 A/m (concave), 6.7 $\times$ 10^5 A/m

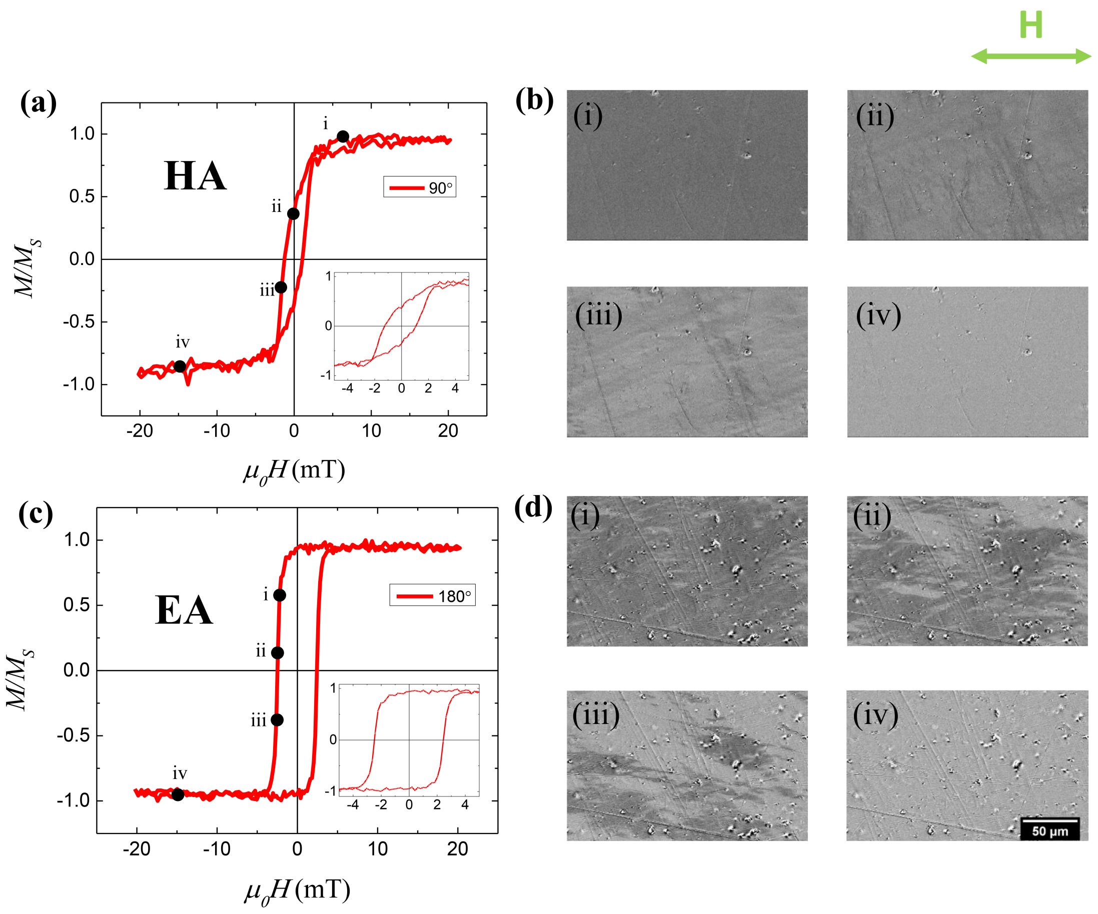

Figure S5: Normalized hysteresis curves of FeGa (28 nm)/Kapton along the (a)HA and (c)EA. MOKE images of magnetic domains corresponding to the HA and EA are represented by (b) and (d) with (i)-(iv) referring the points marked in the hysteresis curves.

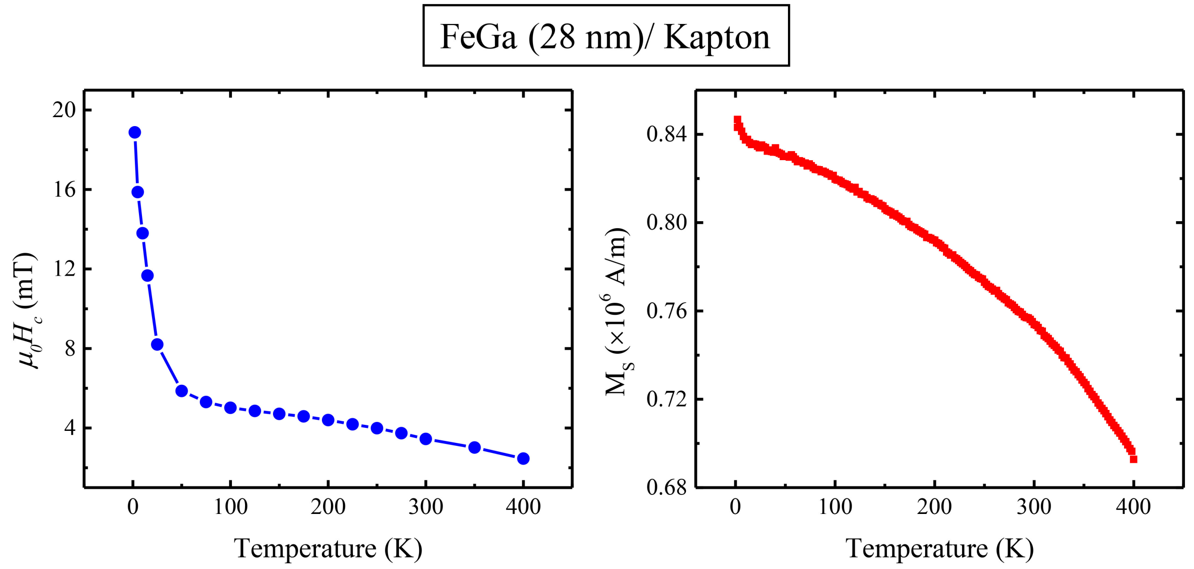

Figure S6: (a) Plot of $\mu_0 H_c$ vs temperature and (b) M_s vs temperature for FeGa (28 nm)/Kapton.

(flat) and 4.6 $\times$ 10^5 A/m (convex). The value of remanent magnetization decreases for the convex state and increases with the concave state as compared to the stress-free state (flat state). Similar trend is observed for the hysteresis at lower temperatures. The coercivity also increases at lower temperatures due to low thermal agitation.

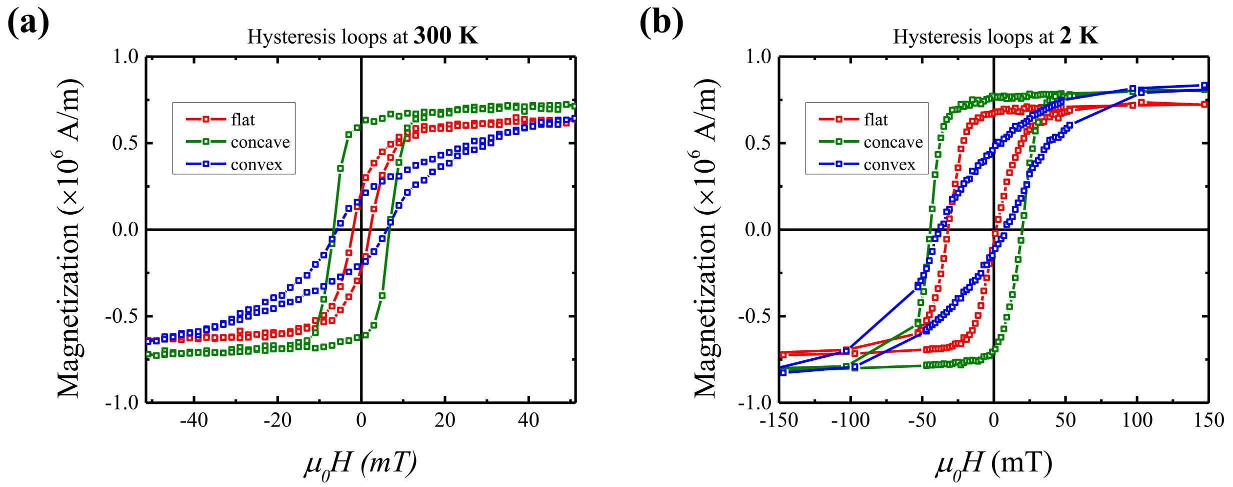

Figure S7: Magnetic hysteresis loops measured with SQUID of FeGa (20 nm)/Kapton sample for flat, concave and convex states, at room temperature (300 K) and at low temperature (2 K) shown by (a) and (b), respectively.
